# Clinical implication of intra-aortic balloon pumping in patients with Takotsubo syndrome and cardiogenic shock

**DOI:** 10.1093/ehjopen/oead050

**Published:** 2023-05-26

**Authors:** Teruhiko Imamura

**Affiliations:** Second Department of Internal Medicine, University of Toyama, 2630 Sugitani Toyama, Toyama 930-0194, Japan

**Keywords:** Heart failure, Mechanical circulatory support, Hemodynamics

**Commentary on Santoro *et al.*, Impact of intra-aortic balloon counterpulsation on all-cause mortality among patients with Takotsubo syndrome complicated by cardiogenic shock: results from the German-Italian-Spanish (GEIST) registry. *Eur Heart J Open* 2023;3:oead003.**

## To editor

The exact mechanism and therapeutic strategy of Takotsubo syndrome remain controversial. Santoro and colleagues compared baseline characteristics and clinical outcomes in patients with Takotsubo syndrome and cardiogenic shock with and without intra-aortic balloon pumping (IABP) using a multicenter international registry of Takotsubo syndrome.^1^ The authors demonstrated that the overall mortality in patients with Takotsubo syndrome and cardiogenic shock was 35%, which was not significantly different between those who received IABP and those who did not. Several concerns have been raised to improve their results.

In their study, key baseline characteristics, such as demographics and comorbidity data, were not significantly different between those with and without IABP.^1^ Nevertheless, there may still be a possibility that IABP was used in sicker patients. Could the authors present more detailed clinical data including laboratory, echocardiographic, and hemodynamic data?

Recently, there has been an increase in the use of other mechanical circulatory support devices, including Impella, to treat patients with Takotsubo syndrome and cardiogenic shock, rather than IABP. Are there any patients in your registry who have received Impella therapy? Theoretically, Impella can bypass left ventricular outflow obstruction, which is often complicated in patients with Takotsubo syndrome and preserve hemodynamics, by forcing blood from the left ventricle into the ascending aorta.^2^ IABP may actually worsen left ventricular outflow obstruction by reducing afterload. In their study, mortality was particularly high in the first 10 days, regardless of the use of IABP.^1^ If the primary cause of death is heart failure, further efforts to stabilize hemodynamics should be made.

The use of IABP did not have a statistically significant effect on long-term survival.^1^ Why do the authors believe that prescribing of beta-blockers improves long-term survival? The international Takotsubo registry showed no significant effect of beta-blocker therapy in suppressing the recurrence of Takotsubo syndrome.^3^

## Data Availability

No new data were generated in support of the article.
